# Increased Circulating Levels of CRP and IL-6 and Decreased Frequencies of T and B Lymphocyte Subsets Are Associated With Immune-Related Adverse Events During Combination Therapy With PD-1 Inhibitors for Liver Cancer

**DOI:** 10.3389/fonc.2022.906824

**Published:** 2022-06-08

**Authors:** Yingying Yu, Siyu Wang, Nan Su, Shida Pan, Bo Tu, Jinfang Zhao, Yingjuan Shen, Qin Qiu, Xiaomeng Liu, Junqing Luan, Fu-Sheng Wang, Fanping Meng, Ming Shi

**Affiliations:** ^1^ 302 Clinical Medical School, Peking University, Beijing, China; ^2^ Senior Department of Infectious Diseases, The Fifth Medical Center of Chinese People's Liberation Army (PLA) General Hospital, Beijing, China; ^3^ The Second School of Clinical Medicine, Southern Medical University, Guangzhou, China; ^4^ Medical School of Chinese People's Liberation Army (PLA), Beijing, China

**Keywords:** liver cancer, immune related adverse events, C-reactive protein, interleukin-6, lymphocyte subsets

## Abstract

**Background:**

Programmed cell death protein 1/programmed death-ligand 1 (PD-1/PD-L1) immune-related adverse events (irAEs) are inevitable in patients with liver cancer. Although the incidence of severe irAEs is low, but can result in fatal consequences. To date, only a few commonly used clinical biomarkers have been reported.

**Aim:**

To assess commonly used clinical biomarkers associated with the occurrence of irAEs to enable better management of irAEs by clinicians.

**Methods:**

We retrospectively reviewed patients with liver cancer treated with at least one cycle of PD-1 immune checkpoint inhibitors (ICIs) combined with tyrosine kinase inhibitors (TKIs). IrAEs were documented according to the common terminology criteria for adverse events version 5. Clinical and laboratory parameters were also evaluated.

**Results:**

A total of 67 patients were included, 36 with irAEs and 31 without irAEs. A total of 104 adverse events occurred; 83 of these events were grade 1/2 (G1/G2), 21 were grade 3/4 (G3/G4), and one died of G4 hepatitis. Patients with irAEs had higher levels of C-reactive protein (CRP) and interleukin-6 (IL-6) and lower levels of lymphocyte subsets, except natural killer (NK) cell counts, than those without irAEs (*P <*0.05). Patients who experienced G3/G4 irAEs had higher levels of CRP and IL-6 and lower levels of CD4+ T lymphocytes and B lymphocytes than those who experienced G1/G2 irAEs (*P <0.05*). Of note, impairments in liver function and routine blood tests were also observed *(P <0.05)*. The results of univariate and multivariate analyses for any grade of irAEs revealed that the combination of sintilimab and lenvatinib (*P=* 0.004, odds ratio [OR]: 7.414, 95% confidence interval [95% CI]: 1.925–28.560) and CRP ≥8.2 mg/L (*P=* 0.024, OR: 3.727, CI: 1.185–11.726) were independent risk factors. Univariate and multivariate analyses of the risk factors of G3/G4 irAEs suggested that the combination of sintilimab and lenvatinib was a potential risk factor (*P* = 0.049, OR: 8.242, CI: 1.006–67.532).

**Conclusion:**

Changes in patient CRP, IL-6, and lymphocyte subsets were associated with irAE onset and may act as potential biomarkers of irAEs. Impairments in liver function and routine blood tests owing to the occurrence of irAEs may become new concerns for clinicians.

## Introduction

Since the discovery of programmed cell death protein 1 (PD-1) in 1992, immunotherapy with antagonistic antibodies targeting the immune checkpoint of PD-1 and its ligand, programmed death-ligand 1 (PD-L1), has prompted a shift in cancer treatment (1, 2). Nearly 800 clinical studies, which have had some remarkably positive results, have been conducted on more than 20 solid and hematological tumors since the approval of pembrolizumab, an anti-PD-1 antibody, in September 2014 for the treatment of advanced melanoma (3). However, the objective response rates for monotherapy, which generally range from 10–30%, are not promising (4–6). Considering the low tumor response to monotherapy, combination therapies are currently at the forefront of treatment. Currently, other immune checkpoint inhibitors (ICIs), tyrosine kinase inhibitors (TKIs), chemotherapy, and radiotherapy are the common combination options that lead to better tumor responses than monotherapy (7, 8). In primary liver cancer, PD-1/PD-L1 ICI monotherapy is mainly used as a second-line treatment (9–11). Owing to the longer progression-free and overall survival of patients using these drugs, the combination therapies currently approved for the first-line treatment of primary liver cancer include nivolumab plus ipilimumab, atezolizumab plus bevacizumab, and pembrolizumab plus lenvatinib (12–14).

Safety is a major concern in the clinical use of PD-1/PD-L1 ICIs. Owing to the widespread use of these agents in clinical practice, unique treatment-specific toxicities, called immune-related adverse events (irAEs), have been reported (15). During anti-PD-1/PD-L1 antibody treatment, irAEs can occur in patients with various types of tumors involving any organ and tissue. These irAEs are generally treatable and have a low incidence and severity (16). Among patients that receive monotherapy, approximately 70% experience at least one adverse event of any grade, 14% experience severe adverse events (grade 3 or 4), and 0.5% experience adverse events that result in death (17). Patients receiving PD-1 antibody monotherapy experience worse irAEs than those receiving PD-L1 agents (18). The overall incidence of adverse events of any grade is nearly 98% for patients treated with combination therapy. Further, the occurrence of grade 3 or 4 irAEs ranges from 10–70%, which is greater than that of patients treated with monotherapy (19).

Elucidating the mechanisms and risk factors of irAEs is valuable for achieving better management. Several factors have been demonstrated to contribute to the mechanisms of PD-1/PD-L1 irAEs, including the disturbance of self-tolerance, cross-reactivity of tumor cell antigens, release of cytokines/chemokines, and enrichment of the microbiome (20). These results are usually determined by analyzing the differences in experimental results between baseline and regular intervals during treatment (21, 22). However, the changes in some indicators when the adverse event occurrence have not been clarified. Further, damage to the corresponding target organs has been the primary focus. Nevertheless, no reports have revealed the changes in non-specific clinical features that simultaneously occur with specific target organ damage.

Identifying the risk factors of irAEs will help with the prompt exclusion of patients who cannot tolerate treatment-related toxicities induced by these treatments, avoid unnecessary pain, and save health care costs. Several factors have been proven to be potential risk factors for irAEs, such as a family history of autoimmune diseases, body mass index (BMI) ≥30, the female gender, and old age (23–30). Some cytokines, including interleukin (IL)-17, IL-2, IL-1, interferon-γ, and Regulated on Activation Normal T cell Expressed and Secreted, have also been demonstrated as risk factors of irAEs. In fact, many of these cytokines have been implicated in the inflammation underlying autoimmune diseases (21, 31–34). C-reactive protein (CRP) and IL-6, which are commonly used clinical indicators of inflammation and autoimmune diseases, have been proven to be associated with the occurrence of irAEs in melanoma and non-small cell lung cancer patients; however, the changes in the levels of these factors in liver cancer patients who experienced irAEs are unclear (35–39). Furthermore, whether an adverse event is an irAE must be determined. In patients experiencing rare or complicated adverse events, evaluating the clinical use of the drug administered and easily observed markers, rather than solely relying on specific target organ lesions and physician experience, will aid in a suitable diagnosis.

In the present study, we focused on the correlation between clinically used peripheral indicators and irAE onset to determine the auxiliary diagnostic indicators that help clinicians identify the occurrence of irAE.

## Materials and Methods

### Patient Inclusion

From March 2020 to July 2021, patients who were diagnosed with liver cancer, including hepatocellular carcinoma and intrahepatic cholangiocarcinoma, through histological or imaging examination and treated with at least one cycle of PD-1 ICIs combined with TKIs, were retrospectively reviewed. All patients visited the Department of Infectious Diseases, The Fifth Medical Center of Chinese PLA General Hospital. PD-1 antibodies, including sintilimab (Innovent Biologics and Eli Lilly and Company), camrelizumab (Jiangsu Hengrui Medicine Co., Ltd), and nivolumab (Bristol–Myers Squibb Co.), were administered at a dose of 200 mg every 3 weeks; 240 mg of toripalimab was administered every 3 weeks. Three available TKIs were used: 400 mg/day of sorafenib (Bayer Schering Pharma AG); 8 to 12 mg/day of lenvatinib (Eisai Co., Ltd.) according to body weight; and 80 mg/day of regorafenib (Bayer Schering Pharma AG). Patients eligible for treatment with transcatheter arterial chemoembolization (TACE) jointly decided on treatments with their physicians, before or after systemic combination therapies. Patients with hepatitis B virus (HBV) infection-related liver cancer received antiviral treatment. IrAEs were documented according to the Common Terminology Criteria for Adverse Events version 5. Spontaneous bacterial peritonitis, a complication of primary liver cancer, was excluded as an irAE. Depending on the toxicity grade, patients who developed irAEs associated with TKI treatment had their dose reduced, suspended, or discontinued, or were administered other TKIs. Based on the severity, patients who developed irAEs associated with PD-1 ICI treatment had their dose suspended or discontinued and were administered immunosuppressive agents.

The date of the follow-up cutoff was October 31, 2021. All data were obtained from patient medical records. The baseline data included patient demographics, Child–Pugh stage, Barcelona Clinic Liver Cancer stage, serum alpha-fetoprotein level, type of combination therapy, and history of TACE. According to the occurrence of irAEs, patients were divided into two groups: irAE and non-irAE. In the irAE group, the following data were collected: levels of CRP, IL-6, lymphocyte subsets, liver function parameters, and routine blood parameters at baseline, irAE onset, and irAE resolution. In the non-irAE group, the above indicators were screened out at both the baseline and follow-up cut-off points. Serum CRP and liver function parameters were measured using the automatic biochemical analyzer, AU5400 (Beckman Coulter Inc., Brea, CA); the upper limit of the normal value for serum CRP was set at 8.2 mg/L. Serum IL-6 was measured using the Roche cobas 8000 (Roche Diagnostics GmbH, Mannheim, Germany); the upper limit of the normal was set at 7 pg/mL. Lymphocyte subsets and counts were measured using the BD FACSCalibur flow cytometer (BD Biosciences, Becton, NJ). The routine blood parameters were measured using the automatic hematology analyzer, HN-2000 series (SYSMEX, Kobe, Japan).

### Statistical Analysis

All statistical analysis were conducted using the IBM SPSS Statistics software application version 25.0 (IBM, Armonk, NY, U.S.A.). Baseline data and adverse events were summarized using descriptive statistics. Continuous variables with normal distribution are presented as mean ± SD; data with non-normal distribution are presented as medians (quartiles); and categorical variables are presented as numbers (percentages). A chi-square or Fisher’s exact test was used to compare the categorical data. Comparisons between the two groups were performed using the t test or nonparametric Mann–Whitney U test, while comparisons within groups were analyzed using the paired t test or nonparametric Wilcoxon paired test. Risk factors associated with irAE development were identified using univariate and multivariate logistic regression analyses. The predictive accuracy of the model was assessed using the Hosmer–Lemeshow test and a receiver operating characteristic curve (ROC). All figures were generated using GraphPad Prism statistical software version 9.0 (GraphPad Software Inc., San Diego, CA). *P*<0.05 was considered statistically significant.

## Results

### Patient Characteristics

We enrolled 67 patients with advanced primary liver cancer and treated them with PD-1 ICIs combined with TKIs from March 2020 to July 2021. Of these patients, 36 were assigned to the irAE group and 31 were assigned to the non-irAE group. During data cut-off, no statistical difference was found between the median follow-up days of the two groups [180.5 (106.8, 348.5) vs. 219.0 (129.0, 385.0), *P* = 0.355]. Patient demographics and the baseline levels of liver function, CRP, IL-6, and lymphocyte subsets are presented in **Table 1**. Compared to patients in the non-irAE group, more patients in the irAE group received a combination of sintilimab and lenvatinib (*P*= 0.004).

**Table 1:** Comparison of baseline characteristics between patients with and without irAEs.

	Total (n=67)	IrAEs (n=36)	Non-irAEs (n=31)	*P* value
Age (y)	57.2 ± 9.7	57.9 ± 9.7	56.4 ± 9.9	0.529
Sex				0.168
Male	57 (85.1%)	33 (91.7%)	24 (77.4%)	
Female	10 (14.9%)	3 (8.3%)	7 (22.6%)	
Types of tumors				0.413
Hepatocellular carcinoma	58 (86.6%)	32 (88.9%)	27 (87.1%)	
Cholangiocarcinoma	8 (13.4%)	4 (11.1%)	4 (12.9%)	
Child-Pugh stage				0.808
A	34 (50.7%)	19 (52.8%)	15 (48.4%)	
B	33 (49.3%)	17 (47.2%)	16 (51.6%)	
BCLC stage				0.320
B	14 (20.9%)	8 (22.2%)	6 (19.4%)	
C (PVTT)	29 (43.3%)	18 (50%)	11 (35.5%)	
C (M)	24 (35.8%)	10 (27.8%)	14 (45.1%)	
Combination treatment				**0.004**
Sintilimab+Lenvatinib	50 (74.6%)	32 (88.9%)	18 (58.1%)	
Sintilimab+Sorafenib	6 (9.0%)	2 (5.6%)	4 (12.9%)	
Camrelizumab+Lenvatinib	4 (6%)	1 (2.7%)	3 (9.7%)	
Nivolumab+Lenvatinib	1 (1.9%)	0	1 (3.2%)	
Toripalimab+Lenvatinib	6 (9%)	1 (2.7%)	5 (16.1%)	
TACE treatment	14 (20.9%)	9 (25%)	5 (16.1%)	0.161
Combination treatment as systemic				0.117
First line	35 (52.2%)	22 (63.1%)	13 (41.9%)	
Second line	9 (13.4%)	6 (16.7%)	3 (9.7%)	
Third line	17 (25.4%)	6 (16.7%)	11 (35.5%)	
Fourth line	6 (9%)	2 (5.5%)	4 (12.9%)	
AFP				0.809
<400 (IU/ml)	41 (61.2%)	22 (61.1%)	19 (61.3%)	
≥400 (IU/ml)	26 (38.8%)	14 (38.9%)	12 (38.7%)	
CRP (mg/L)	11.6 (4.3,29.0)	13.6 (6.2,33.9)	7.6 (3.7, 23.4)	0.809
IL-6 (pg/mL)	18.0 (6.6, 25.6)	18.4 (6.9, 27.1)	12.1 (6.0, 22.7)	0.135
Total lymphocyte (count/μL)	1260.5 (799.3, 1569.0)	1262.0 (799.5, 1569.0)	1158.0 (799.0, 1575.0)	0.341
T lymphocyte (count/μL)	883.0 (580.3, 1234.5)	941.0 (546.0, 1341.0)	814.0 (592.5, 1220.0)	0.662
CD4 T lymphocyte (count/μL)	477.5 (295.0, 717.0)	467.0 (272.5, 725.0)	485.0 (309.5, 719.5)	0.938
CD8 T lymphocyte (count/μL)	347.0 (169.0, 448.5)	329.0 (163.0, 440.5)	348.0 (208.0, 457.0)	0.652
B lymphocyte (count/μL)	114.5 (57.5, 303.5)	107.0 (54.5, 216.5)	126.0 (59.0, 199.0)	0.577
NK cell (count/μL)	152.0 (84.3, 217.8)	160.0 (83.5, 251.0)	130.0 (88.0, 199.0)	0.505
CD4/CD8	1.5 (1.1, 2.0)	1.5 (1.0, 2.0)	1.6 (1.1, 2.1)	0.485
ALB (g/L)	35.0 (31.0, 38.0)	33.0 (31, 38.6)	36.0 (31.0, 38.0)	0.671
TBIL (umol/L)	21.6 (15.0, 31.0)	21.8 (15.3, 33.8)	21.3 (13.5, 25.5)	0.401
ALT (U/L)	36.0 (23.0, 63.0)	41.5 (24.3, 64.5)	35.0 (22.0, 49.0)	0.206
CHE (U/L)	4141.0 (3184.0, 5888.0)	4026.5 (2796.0, 5532.0)	4035.0 (3128.0, 6253.0)	0.487
LDH (U/L)	243.0 (187.0, 287.0)	234. 0 (188.8, 273.5)	253.0 (185.0, 294.0)	0.578
PTA (%)	71.3 (58.6, 84.9)	71.8 (58.6, 86.3)	70.4 (60.1, 81.2)	0.668
WBC (10^9/L)	4.7 (3.2, 6.7)	4.6 (3.4, 6.6)	4.9 (2.9, 6.8)	0.862
ANC (10^9/L)	3.1 (1.7, 4.2)	3.1 (1.8, 4.1)	3.1 (1.7, 4.5)	0.945
LYM (10^9/L)	1.1 (0.8, 1.6)	1.0 (0.78, 1.5)	1.2 (0.8, 1.6)	0.397
AMC (10^9/L)	0.4 (0.2, 0.5)	0.4 (0.2, 0.5)	0.4 (0.2, 0.6)	0.780
PLT (10^9/L)	124.0 (100.0, 142.0)	124.0 (98.5, 205.8)	120.0 (81.0, 192.0)	0.543
NLR	2.6 (1.9, 3.8)	2.53 (1.76,4.33)	2.77 (2.00, 3.81)	0.870
PLR	121.5 (76.5, 157.9)	122.1 (74.9, 179.8)	121.5 (81.7, 157.7)	0.980
PWR	26.6 (20.6, 36.1)	26.50 (20.63, 31.16)	26.93 (20.97, 41.03)	0.651

BCLC, Barcelona Clinic Liver Cancer; M, metastasis; PVTT, portal vein tumor thrombus; TACE, transcatheter arterial chemoembolization; AFP, Alpha‐Fetoprotein; CRP, C-reactive protein; IL-6, interleukin-6;ALB, albumin; TBIL, total bilirubin; ALT, alanine aminotransferase; CHE, cholinesterase; LDH, lactate dehydrogenase; PTA, prothrombin activity; WBC, white blood cell; ANC, absolute neutrophil count; LYM, absolute lymphocytes; AMC, absolute monocyte count; PLT, platelets; NLR, neutrophil-lymphocyte ratio; PLR, platelet -lymphocyte ratio; PWR, platelet-white blood cell ratio. The p-value of the significance shown in bold in the table.

### Occurrence of irAEs

Among the enrolled patients, 36 (53.7%) experienced at least one adverse event of any grade, and 17 (25.4%) developed grade 3 (G3)/grade 4 (G4) adverse events. All patients who experienced severe irAEs received glucocorticoids according to clinical guidelines. The overall occurrence of adverse events in patients is shown in **Table 2**. The most common mild adverse effects were fever (n = 13, 19.4%), hypertension (n = 10, 14.9%), and fatigue (n = 10, 14.9%); hepatitis (n = 7, 10.4%) was the most common event among G3/G4 irAEs. Most patients with G3/G4 adverse events had improvements with glucocorticoid therapy. Only one patient died as a result of G4 hepatitis.

**Table 2:** Overall incidence of adverse events.

N=76	G1/G2	G3/4
Fever	13 (19.4%)	0
Fatigue	12 (17.9%)	0
Hypertension	10 (14.9%)	0
Rash	8 (11.9%)	2 (3.0%)
Hypothyroidism	8 (11.9%)	0
Diarrhea	7 (10.4%)	2 (3.0%)
hepatitis	1 (1.5%)	7 (10.4%)
Pneumonia	1 (1.5%)	1 (1.5%)
Lymphopenia	3 (4.5%)	0
Pruritus	3 (4.5%)	0
Proteinuria	3 (4.5%)	0
Renal dysfunction	3 (4.5%)	1 (1.5%)
Thrombocytopenia	2 (3%)	2 (3%)
Weight loss	2 (3%)	0
Hyperthyroidism	2 (3%)	0
Cardiotoxicity	3 (4.5%)	0
Hyperglycemia	1 (1.5%)	0
Arthritis	1 (1.5%)	0
Bacterial infection	0	3 (4.5%)
fungal infection	0	1 (1.5%)
Herpes virus infection	0	1 (1.5%)
Intestinal infections	0	1 (1.5%)

### Levels of CRP, IL-6, and Lymphocyte Subsets Associated With irAEs

To determine the correlation between the occurrence of irAEs and the levels of CRP, IL-6, and lymphocyte subsets, we focused on irAEs that occurred during hospitalization. In the present study, 40 adverse events were identified in 36 patients, 17 of which were G3/G4 irAEs (**Table S1**).

**Figure 1:**
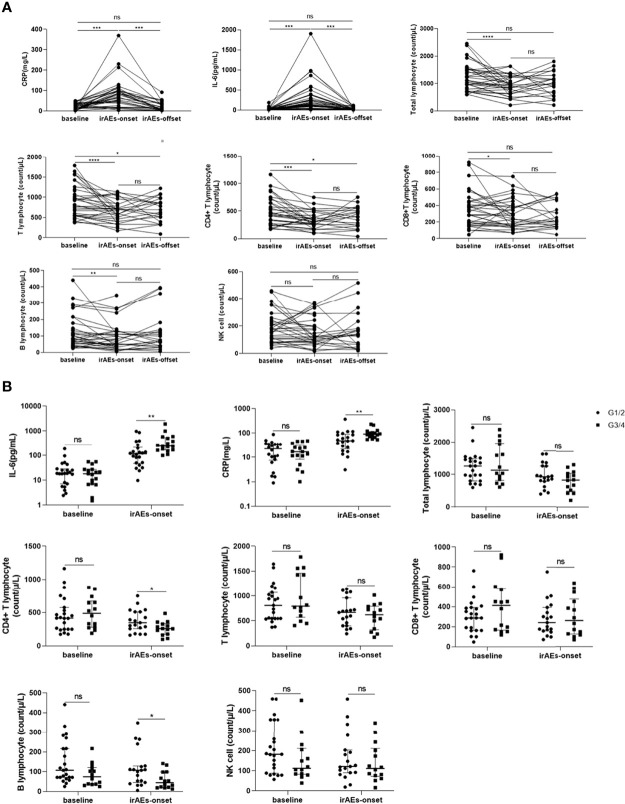
Changes in c-reactive protein (CRP) and interleukin-6 (IL-6) levels with the development of immune-related adverse events (irAEs). **(A)** Changes inn CRP, IL-6, and lymphocyte subsets at baseline, irAEs-onset, and irAEs-offset in patients with irAEs. **(B)** Comparison of CRP and IL-6 levels between baseline and irAEs onset in patients with different grades of irAEs. CRP, C-reactive protein; IL-6, interleukin-6; G1: grade 1; G2, grade 2; G3, grade 3; G4, grade 4; *P < 0.05; **P < 0.01; ***P < 0.001; ****P < 0.000; ns, no statistical difference.

First, the baseline levels of CRP, IL-6, and lymphocyte subsets were compared between the irAE and non-irAE groups, and no statistical difference was found (*P*>0.05) (**Table 1**). In patients with irAEs, upon irAE onset, the CRP and IL-6 counts increased (*P*<0.05); the total lymphocyte, T lymphocyte, CD4+ T lymphocyte, CD8+ T lymphocyte, and B lymphocyte counts decreased (*P*<0.05), and the NK cell count did not change (*P*>0.05). When patients recovered, their CRP and IL-6 levels returned to baseline; however, the lymphocyte subsets did not return to baseline (**Figure 1A**). In patients without irAEs, no significant differences were found in the levels of the above parameters between the baseline and follow-up cut-off points (**Figure S1**).

**Figure 2:**
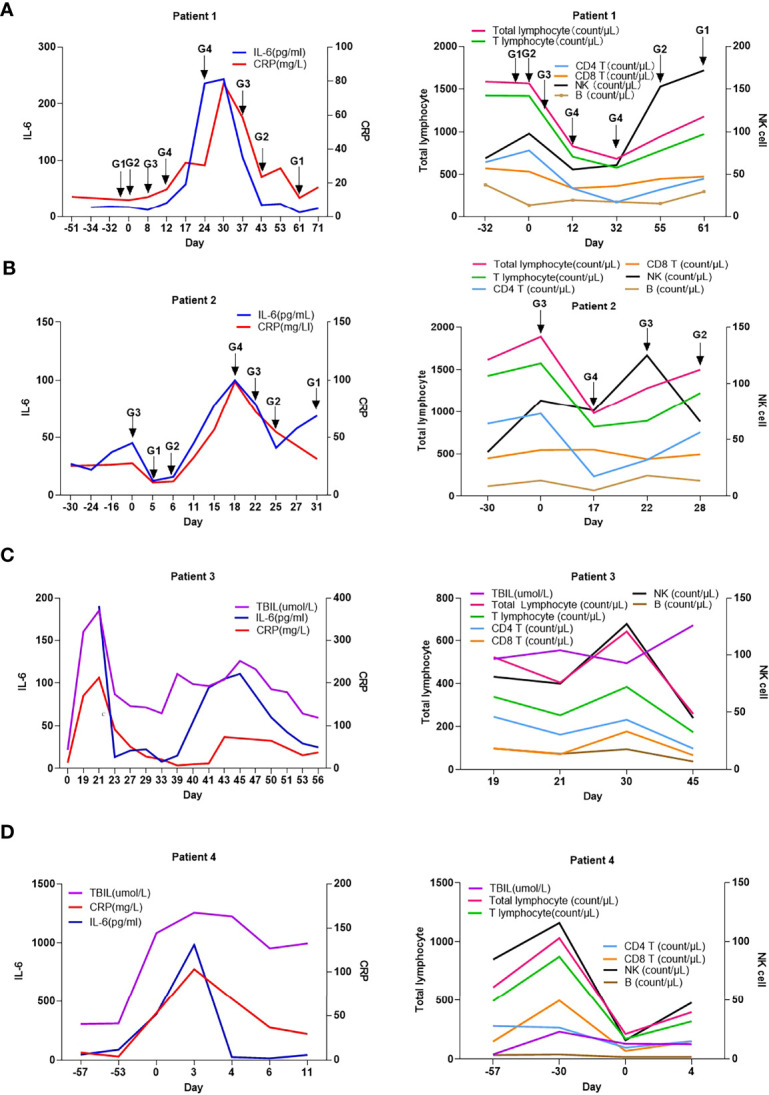
Changes in c-reactive protein level, interleukin-6 level, and lymphocyte subsets with the development of immune-related adverse events in 4 patients overtime. **(A)** patient 1; **(B)** patient 2; **(C)** patient 3; **(D)** patient 4; CRP, C-reactive protein; IL-6, interleukin-6; G1: grade 1; G2, grade 2; G3, grade 3; G4, grade 4; 0 Day, day of readmission.

We proceeded to evaluate the differences in levels of CRP, IL-6, and lymphocyte subsets between patients with G1/G2 and G3/G4 irAEs. Compared to patients with G1/G2 irAEs, those with G3/G4 irAEs had a higher increase in the levels of CRP and IL-6 and a lower decrease in the levels of CD4+ T lymphocytes and B lymphocytes upon irAE onset (*P*<0.05) (**Figure 1B**). Based on these results, we analyzed the CRP, IL-6, and lymphocyte subset levels of a given adverse event at different stages. we selected four patients who experienced G4 irAEs that lasted >1 week; of these patients, two had skin toxicities and two had hepatitis, results suggested that the changes in CRP, IL-6, and lymphocyte subsets were found to be consistent with the severity of irAEs over time (**Figure 2**).

### Liver Function and Routine Blood Value Changes Associated With irAEs

**Figure 3:**
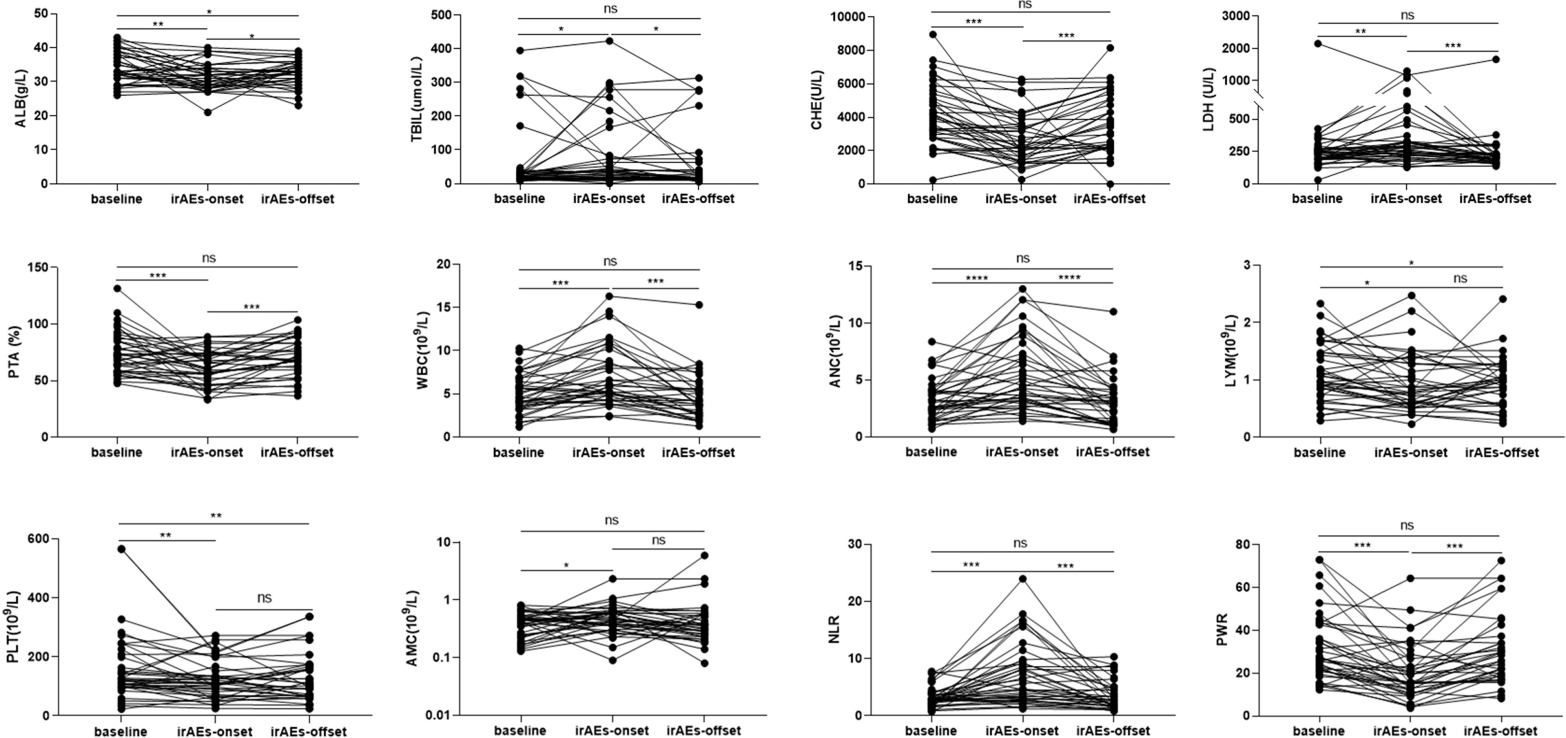
Comparison of the laboratory results at baseline, immune-related adverse event (irAE)- onset, and irAEs-offset in patients with irAEs. ALB, albumin; TBIL, total bilirubin; CHE, cholinesterase; LDH, lactate dehydrogenase; PTA, prothrombin activity; WBC, white blood cell; ANC, absolute neutrophil count; AMC, absolute monocyte count; LYM, absolute lymphocytes; PLT, platelets; NLR, neutrophil-lymphocyte ratio; PWR, platelet-white blood cell ratio. *P < 0.05; **P < 0.01; ***P < 0.001; ****P < 0.0001; ns, no statistical difference.

The liver function and routine blood test results of patients were closely monitored. The baseline data revealed no significant difference between the irAE and non-irAE groups (*P*>0.05) (**Table 1**). In the irAE group, total bilirubin (TBIL), lactate dehydrogenase (LDH), white blood cell count (WBC), absolute neutrophil count (ANC), absolute monocyte count (AMC), and neutrophil-lymphocyte ratio (NLR) increased, while albumin (ALB), cholinesterase (CHE), prothrombin activity (PTA), absolute lymphocytes (LYM), platelets (PLT), and platelet–white blood cell ratio (PWR) decreased (*P*<0.05) (**Figure 3**). As eight patients had hepatitis and displayed a decrease in ALB and CHE and an increase in ALT and TBIL, which might lead to bias in our results, these patients were excluded and a re-analysis was performed. However, similar results were obtained (**Figure S2**). In the non-irAE group, the data between the baseline and follow-up cut-off points showed no significant difference (**Table S2**).

### Univariate and Multivariate Analysis of the Risk Factors for irAE

Many clinicians seek to identify indicators that can predict the occurrence of irAEs. Based on the above results, we conducted univariate and multivariate binary logistic regression analyses to evaluate the risk factors associated with any irAE grade, especially G3/G4.

The risk factors for irAEs of any grade were analyzed. First, the correlation between the occurrence of irAEs and the baseline characteristics of patients was determined. Univariate analysis revealed that patients treated with the combination of sintilimab and lenvatinib had a higher probability of developing irAEs of any grade (*P* = 0.003) (**Table S3**).

**Figure 4:**
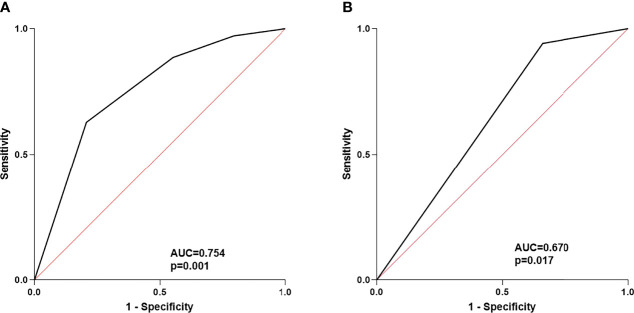
**(A)** ROC curve of the risk factors for any grade of irAEs; **(B)** ROC curve of risk factors for 3/4 grade of irAEs.

Second, we assessed the correlation between the baseline levels of CRP, IL-6, liver function parameters, routine blood parameters, and adverse events. Based on the continuous variables, all parameters were divided into two groups according to normal or median values. Univariate analysis revealed that patients with CRP ≥8.2 mg/L (*P =* 0.033) and IL-6 ≥18 pg/mL (*P =* 0.027) had a significantly higher incidence of irAEs of any grade (**Table S3**). Multivariate analysis confirmed that CRP ≥8.2 mg/L (*P* = 0.024, odds ratio [OR]: 3.727, 95% confidence interval [95% CI]: 1.185–11.726) and the combination of sintilimab and lenvatinib (*P* = 0.004, OR: 7.414, 95% CI: 1.925–28.560) were independent risk factors for irAEs of any grade (**Table 3**). The area under the ROC curve was used to assess the model. Based on the results, the model had good predictive accuracy (*P* = 0.001, AUC = 0.754, 95% CI: 0.633–0.875), with a significant difference from that of the reference line (**Figure 4A**). The Hosmer–Lemeshow test (chi-square: 0.103, degree of freedom = 2, *P* = 0.950) also revealed that the model was good at predicting whether patients would develop irAEs of any grade.

**Table 3:** Multivariate binary logistic regression analysis for risk factors of all grade and grade3/grade4 irAEs.

	Multivariate analysis for all grade irAEs			Multivariate analysis for grade3/grade4 irAEs		
	*P* value	Odds ratio	95% CI	*P* value	Odds ratio	95% CI
Treatment						
Other combination treatment	1.000	–	–	1.000	–	–
Sintilimab+Lenvatinib	**0.004**	7.414	1.925-28.560	**0.049**	8.242	1.006-67.532
CRP						
<8.2(mg/L)	1.000	–	–			
≥8.2(mg/L)	**0.024**	3.727	1.185-11.726			
IL-6						
<18(pg/mL)	1.000	–	–			
≥18(pg/mL)	0.302	2.167	0.498-9.425			

Other treatment, including Sintilimab+Sorafenib, Camrelizumab+Lenvatinib, Nivolumab+Lenvatinib, Toripalimab+Lenvatinib; CRP, C-reactive protein; IL-6, interleukin-6. The p-value of the significance shown in bold in the table.

Third, we analyzed the risk factors of G3/G4 irAEs. The results of univariate and multivariate analyses suggested that the combination of sintilimab and lenvatinib was a potential biomarker for G3/G4 irAEs (*P* = 0.049, OR: 8.242, CI: 1.006–67.532) (**Table 3**). Of note, the ROC curve demonstrated a moderate predictive accuracy for this model (*P* = 0.017, AUC = 0.670, 95% CI: 0.537–0.803) (**Figure 4B**).

## Discussion

In contrast to previous studies that discussed the mechanism of irAEs through basic scientific research, which was difficult to perform, we opted to focus on some parameters that are commonly used in clinical practice. Accordingly, we provided several novel insights into the clinical features of patients with irAEs and the biomarkers and predictors of irAEs.

In this retrospective cohort study, to identify potential biomarkers of irAE development, we analyzed data from 67 patients with primary liver cancer who received PD-1 combination therapy and experienced or did not experience irAEs. First, the occurrence of irAEs was found to be accompanied by changes in immunological parameters, as shown by the increased levels of CRP and IL-6 and the decreased levels of the lymphocyte subsets. Second, patients with irAEs were found to have impaired liver function and abnormal routine blood test results, regardless of the irAE type. Third, the combination of sintilimab and lenvatinib and CRP levels ≥8.2 mg/L, which is the upper limit of normal (ULN), were considered independent risk factors for the development of irAEs of any grade; the combination therapy might also be a potential risk factor for the development of G3/G4 irAEs.

Of the 67 patients in this study, 36 had at least one irAE of any grade, and 17 developed G3/G4 irAEs. The most common G1/G2 irAE was fever, and the most common G3/G4 irAE was hepatitis (10.4%). Among patients with ≥G3 irAEs, seven permanently discontinued the use of ICI therapy, and one died due to a severe irAE. The higher incidence of severe irAEs in our study may be due to the inclusion of patients with Child–Pugh stage B (n = 33, 49.3%). As patients with liver cirrhosis have cirrhosis-associated immune dysfunction, pre-existing hepatitis and extrahepatic symptoms in patients may be difficult to distinguish from irAEs and synergistically act to aggravate organ dysfunction (40). In our study, eight patients treated with sintilimab and lenvatinib developed hepatitis, which was characterized by elevated TBIL, ALT, and AST. Among these patients, one had mild hepatitis, and seven had severe hepatitis; one of the seven patients died from G4 hepatitis. Based on multivariate analysis, the combination of sintilimab and lenvatinib might act as a potential independent risk factor for irAEs of any grade, especially G3/4. Therefore, our data suggest that the occurrence of irAEs of any grade, especially G3/G4 irAEs such as severe hepatitis, should be the focus when patients with liver cancer are treated with sintilimab and lenvatinib to avoid a serious prognosis.

As non-specific biomarkers, the levels of CRP and IL-6 are commonly used to assess the presence and severity of acute and chronic inflammation, infection, tissue damage, and cancer (41–43). CRP and IL-6 levels are also associated with the occurrence of irAEs. Patients with melanoma displayed elevated CRP levels when irAEs occurred, and patients with a CRP level more than twice the ULN were more likely to have irAEs than those with a CRP level less than the ULN (35, 36). In a case report, the baseline CRP level increased from 14.2 to 138.9 mg/L when pneumonitis occurred (37). In this study, we analyzed some biomarkers associated with irAEs. Based on our results, a transient increase occurred in CRP and IL-6 levels upon irAE onset. These increased levels returned to baseline upon resolution of the irAEs. A positive association was found between irAE severity and CRP and IL-6 levels. Multivariate logistic regression analyses suggested that CRP levels ≥8.2 mg, which is the ULN, was a potential independent risk factor of irAE development. Although univariate analysis suggested that baseline IL-6 levels ≥18 pg/mL may be a risk factor of irAEs, the results of multivariate analysis did not indicate statistical significance. In conjunction with IL-1β and tumor necrosis factor-α, IL-6, an upstream cytokine of CRP, promotes the production of CRP in the liver. Therefore, IL-6 has a significantly positive correlation with CRP, which makes the multivariate regression analysis of IL-6 was not statistically significant (44). Our results suggest that CRP and IL-6 could be potential biomarkers of irAEs; however, their origins need further tracing.

The characteristics of the lymphocyte subsets were analyzed. The lymphocyte subset counts, except for the NK cell count, were found to be negatively correlated with the occurrence of irAEs and the grade of irAEs. The mechanism for the decrease in lymphocyte subsets is unclear, but may be related to a systemic inflammatory response caused by a cytokine storm, which has been observed in COVID-19 patients. Compared to patients with mild COVID-19, those with a severe form of the illness would experience a cytokine storm and be characterized by increased levels of CRP, IL-6, granulocyte colony-stimulating factor, interferon-inducible protein-10, monocyte chemotactic protein-1, macrophage inflammatory protein-1α/β, IL-8, and other cytokines, which can promote chemotaxis or the apoptosis of peripheral blood lymphocyte subsets, leading to a decrease in cell number (45–48). Compared with the rapid return of CRP and IL-6 to baseline levels, the lymphocyte subsets did not return to this level; this occurrence may further suggest that the decrease in immune cells may be related to the apoptosis induced by the cytokine storm. Notably, PD-1 is always expressed in T and B cells and less expressed in NK cells (49). When PD-1 antibodies are used, T and B cells are activated, whereas NK cells may be less activated. Thus, when the irAEs occurred, activated T and B lymphocytes were affected by the cytokine storm, whereas NK cells did not undergo apoptosis or chemotaxis, may be the reason for the lack of change in NK cells in patients with irAEs. Nevertheless, further studies are needed on the precise mechanism of the decreased lymphocyte subset count in patients with irAEs.

We determined whether liver function and routine blood test results were associated with irAE occurrence. The irAEs were found to be positively correlated with TBIL, LDH, PT, WBC, ANC, AMC, and NLR, and negatively correlated with ALB, CHE, PTA, LYM, PLT, and PWR. The above results suggest the activation of the body’s innate immunity, which shares the same characteristics as the inflammatory response (50). Although the results are similar to those of the lymphocyte subsets and may be attributed to the release of CRP and IL-6, the precise mechanism for these results is unclear (51). To the best of our knowledge, this is the first study to report the aggravated impairment of liver function in patients with non-hepatitis adverse events. Liver function impairment may be due to lymphocytes converging into the liver under chemotaxis induced by some chemokines, which destroy tumor cells or normal hepatocytes, resulting in liver function impairment. Accordingly, the mechanism of lymphocytic infiltration in the normal liver and tumor tissues is worth investigating.

In our study, to explore the biomarkers and predictors of irAEs during clinical treatment, some clinical parameters of laboratory inspection were analyzed. Based on our results, irAE severity was positively correlated with CRP and IL-6 levels and negatively correlated with lymphocyte subset levels. Further, CRP could be a potential predictor for the early diagnosis of irAEs. The current study is the first to demonstrate the impairments in liver function and blood parameters as characteristics of patients with liver cancer and treatment-adverse events. These findings can help physicians better identify irAEs in these patients and shift their focus to the impairment of liver function and secondary infections caused by decreased immune cells and the target lesions of irAEs.

Our study had several limitations. First, this was a single-center retrospective study with a relatively small sample size. Second, the selected adverse events only occurred during hospitalization; disregarding the changes in the CRP and IL-6 levels of patients with irAEs that occurred outside the hospital may lead to some degree of bias. Therefore, large prospective clinical trials might be needed to further reveal the association between CRP, IL-6, lymphocyte subsets, liver function, routine blood parameters, and irAE, as well as other unidentified markers.

## Data Availability Statement

The raw data supporting the conclusions of this article will be made available by the authors, without undue reservation.

## Ethics Statement

The studies involving human participants were reviewed and approved by Chinese Ethics Committee of Registering Clinical Trials. The patients/participants provided their written informed consent to participate in this study.

## Author Contributions

MS, FM, and F-SW conceived the study, wrote the manuscript and constructed the figures with YY, SW, NS, MS, FM, and F-SW edited the manuscript and provided comments and feedback. SP, BT, YS, QQ, and JL provided the information of the patient. All authors read and approved the final manuscript.

## Funding

This work was supported by grants from Beijing Municipal Science and Technology Commission (Z201100005520047); Ministry of Science and Technology of the People’s Republic of China (2019YFC0840704); the National Natural Science Foundation of China (82070617) and the Innovation Groups of the National Natural Science Foundation of China (81721002).

## Conflict of Interest

The authors declare that the research was conducted in the absence of any commercial or financial relationships that could be construed as a potential conflict of interest.

## Publisher’s Note

All claims expressed in this article are solely those of the authors and do not necessarily represent those of their affiliated organizations, or those of the publisher, the editors and the reviewers. Any product that may be evaluated in this article, or claim that may be made by its manufacturer, is not guaranteed or endorsed by the publisher.

## Supplementary Material

Supplementary Figure 1 Comparison of CRP, IL-6, and lymphocyte subsets between baseline and the follow-up cut-off point in non-irAEs patients. CRP, C-reactive protein; IL-6, interleukin-6; ns, no statistical difference.

Supplementary Figure 2 Comparison of liver function and blood routine at baseline, irAEs onset, and post-irAEs in patients with irAEs, except hepatitis patients. ALB, albumin; TBIL, total bilirubin; CHE, cholinesterase; LDH, lactate dehydrogenase; PTA, prothrombin activity; WBC, white blood cell; ANC, absolute neutrophil count; AMC, absolute monocyte count; LYM, absolute lymphocytes; PLT, platelets; NLR, neutrophil-lymphocyte ratio; PWR, platelet-white blood cell ratio. **P* < 0.05; ***P* < 0.01; ****P* < 0.001; ns, no statistical difference.

Supplementary Table 1 The occurrence of adverse events at patient admission to the hospital.

Supplementary Table 2 Comparison of laboratory results between baseline and the follow-up cut-off in patients without irAEs. ALB, albumin; TBIL, total bilirubin; aminotransferase; CHE, cholinesterase; LDH, lactate dehydrogenase; PTA, prothrombin activity; WBC, white blood cell; ANC, absolute neutrophil count; LYM, absolute lymphocytes; AMC, absolute monocyte count; PLT, platelets; NLR, neutrophil-lymphocyte ratio; PLR, platelet -lymphocyte ratio; PWR, platelet-white blood cell ratio.

Supplementary Table 3 Univariate binary logistic regression analysis for risk factors of all grade and grade3/grade4 irAEs. BCLC, Barcelona Clinic Liver Cancer; M, metastasis; PVTT, portal vein tumor thrombus; TACE, transcatheter arterial chemoembolization; CRP, C-reactive protein; IL-6, interleukin-6;AFP, Alpha‐Fetoprotein; ALB, albumin; TBIL, total bilirubin; ALT, alanine aminotransferase; AST, aspartate aminotransferase; CHE, cholinesterase; LDH, lactate dehydrogenase; PTA, prothrombin activity; WBC, white blood cell; ANC, absolute neutrophil count; LYM, absolute lymphocytes; AMC, absolute monocyte count; PLT, platelets; NLR, neutrophil-lymphocyte ratio; PLR, platelet -lymphocyte ratio; PWR, platelet-white blood cell ratio.
