# Supplementary figures and images for: Increased Circulating Levels of CRP and IL-6 and Decreased Frequencies of T and B Lymphocyte Subsets Are Associated With Immune-Related Adverse Events During Combination Therapy With PD-1 Inhibitors for Liver Cancer
Source: Front Oncol. 2022 Jun 8;12:906824. doi: 10.3389/fonc.2022.906824 (PMC9232255; doi:10.3389/fonc.2022.906824)

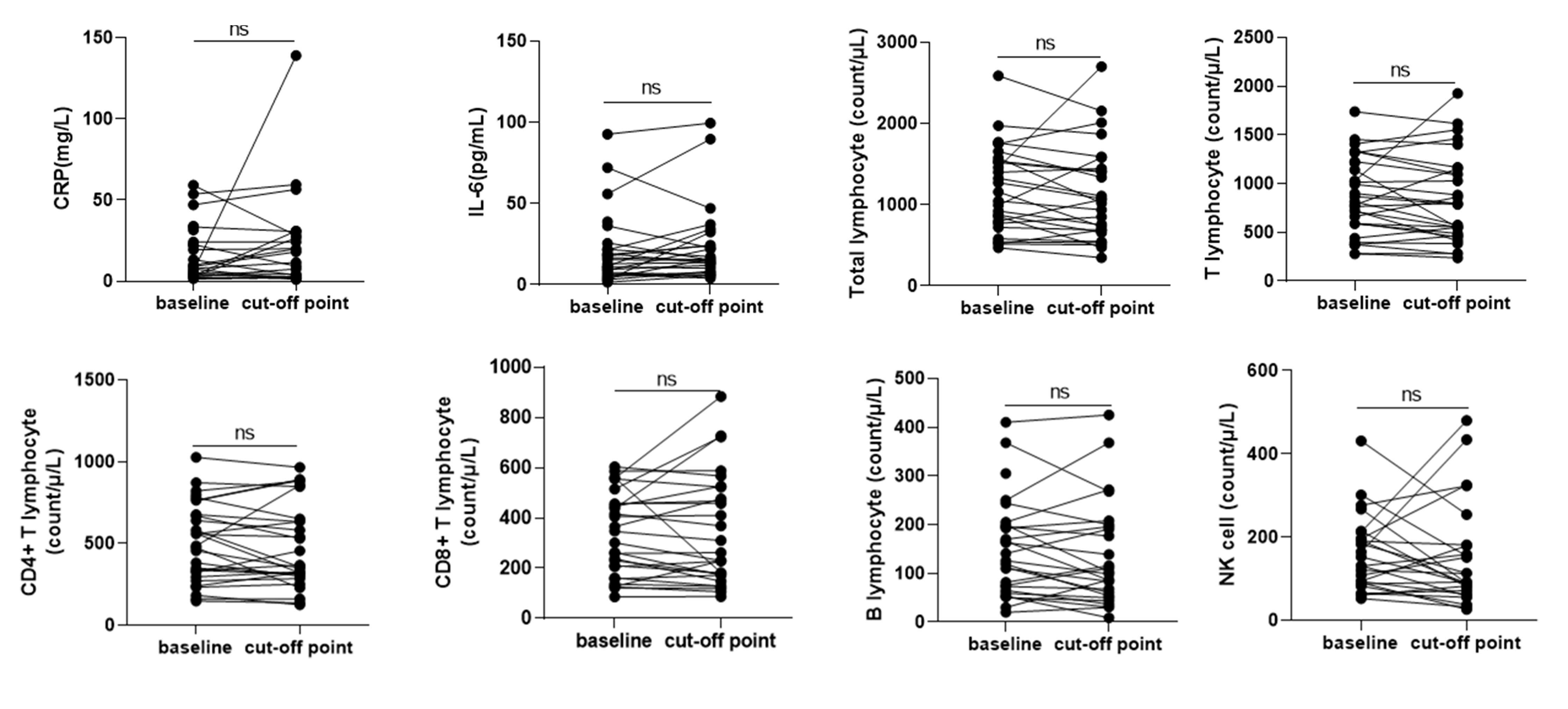

Supplement: Supplementary Figure 1 — Comparison of CRP, IL-6, and lymphocyte subsets between baseline and the follow-up cut-off point in non-irAEs patients. CRP, C-reactive protein; IL-6, interleukin-6; ns, no statistical difference. [file Image_1.png]

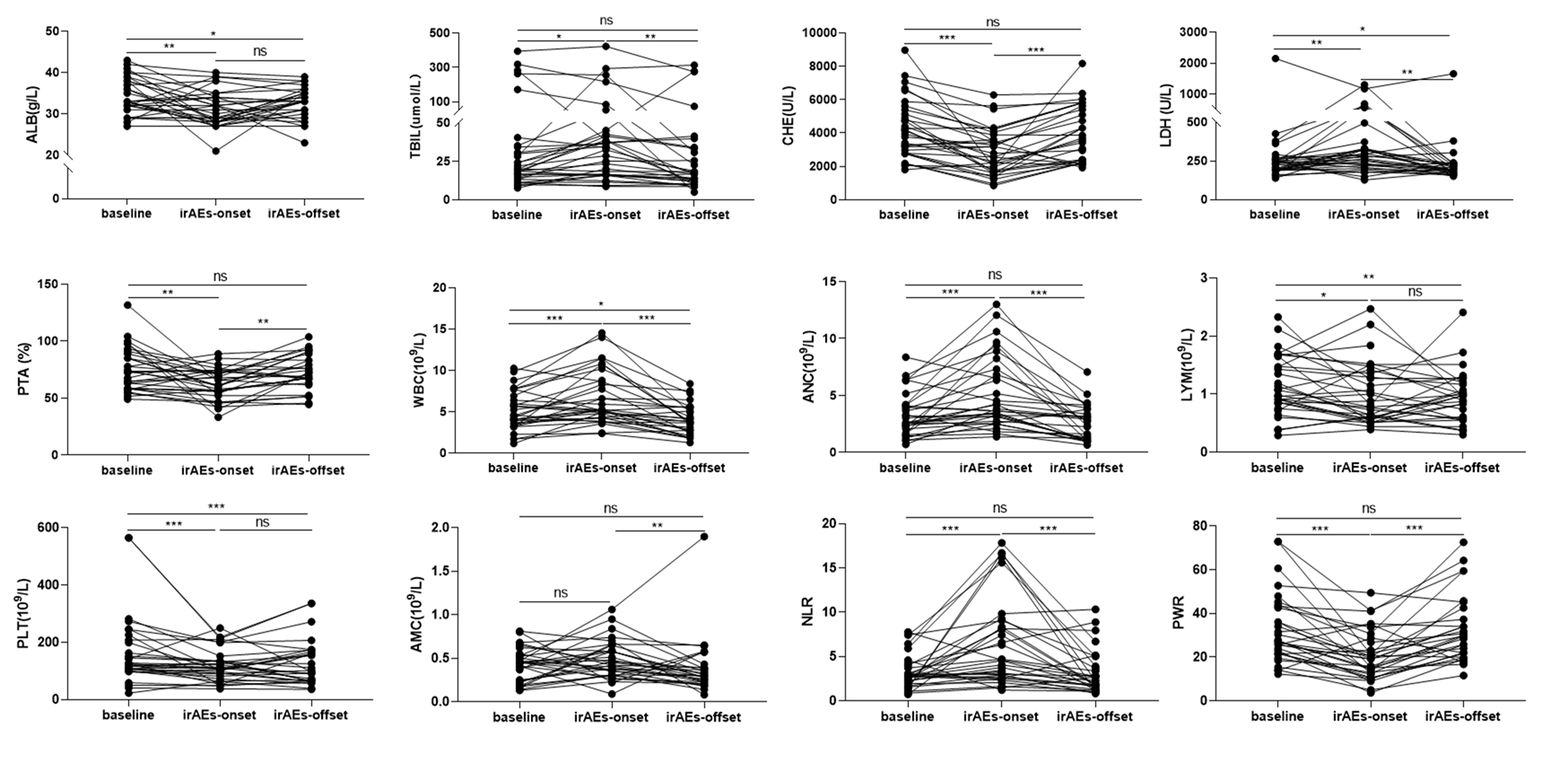

Supplement: Supplementary Figure 2 — Comparison of liver function and blood routine at baseline, irAEs onset, and post-irAEs in patients with irAEs, except hepatitis patients. ALB, albumin; TBIL, total bilirubin; CHE, cholinesterase; LDH, lactate dehydrogenase; PTA, prothrombin activity; WBC, white blood cell; ANC, absolute neutrophil count; AMC, absolute monocyte count; LYM, absolute lymphocytes; PLT, platelets; NLR, neutrophil-lymphocyte ratio; PWR, platelet-white blood cell ratio. *P < 0.05; **P < 0.01; ***P < 0.001; ns, no statistical difference. [file Image_2.png]
