# Supplementary material for: Increased Circulating Levels of CRP and IL-6 and Decreased Frequencies of T and B Lymphocyte Subsets Are Associated With Immune-Related Adverse Events During Combination Therapy With PD-1 Inhibitors for Liver Cancer
Source: Front Oncol. 2022 Jun 8;12:906824. doi: 10.3389/fonc.2022.906824 (PMC9232255; doi:10.3389/fonc.2022.906824)
Supplement: Supplementary Table 1 — The occurrence of adverse events at patient admission to the hospital. [file Table_1.pdf]

Table S1 The occurrence of adverse events when patients admitted in hospital

	**G1/G2**	**G3/G4**
Fever	13	0
Rash	5	2
Hepatitis	1	7
Pneumonia	1	2
Pruritus	1	0
Diarrhea	1	0
Cardiotoxicity	1	0
Bacterial infection	0	3
fungal infection	0	1
Herpes virus infection	0	1
Intestinal infections	0	1
