# Supplementary material for: Increased Circulating Levels of CRP and IL-6 and Decreased Frequencies of T and B Lymphocyte Subsets Are Associated With Immune-Related Adverse Events During Combination Therapy With PD-1 Inhibitors for Liver Cancer
Source: Front Oncol. 2022 Jun 8;12:906824. doi: 10.3389/fonc.2022.906824 (PMC9232255; doi:10.3389/fonc.2022.906824)
Supplement: Supplementary Table 2 — Comparison of laboratory results between baseline and the follow-up cut-off in patients without irAEs. ALB, albumin; TBIL, total bilirubin; aminotransferase; CHE, cholinesterase; LDH, lactate dehydrogenase; PTA, prothrombin activity; WBC, white blood cell; ANC, absolute neutrophil count; LYM, absolute lymphocytes; AMC, absolute monocyte count; PLT, platelets; NLR, neutrophil-lymphocyte ratio; PLR, platelet -lymphocyte ratio; PWR, platelet-white blood cell ratio. [file Table_2.pdf]

Table S2 Comparison of laboratory results between baseline and follow-up cut-off in patients without irAEs.

	**Baseline**	**Follow up cut-off**	**p value**
ALB(g/L）	36.0 (31.0, 38.0)	34.0 (30.0, 36.0）	0.062
TBIL (umol/L)	21.3 (13.5, 25.5)	21.3 (14.6, 27.5)	0.660
CHE (U/L)	4035.0 (3128.0, 6253.0)	3887.0 (2382.0, 5927.0)	0.060
LDH (U/L)	253.0 (185.0, 294.0)	213.0 (186.0, 254.0)	0.552
PTA (%)	70.4 (60.1, 81.2)	71.3 (57.3, 83.9)	0.761
WBC (10^9/L)	4.9 (2.9, 6.8)	4.3 (2.9, 5.9)	0.368
ANC (10^9/L)	3.1 (1.7, 4.5)	2.5 (1.5, 3.7)	0.490
LYM (10^9/L)	1.2 (0.8, 1.6)	0.88 (0.68, 1.5)	0.223
AMC (10^9/L)	0.36 (0.24, 0.60)	0.39 (0.31, 0.57)	0.818
PLT (10^9/L)	120.0 (81.0, 192.0)	122.5 (67.0,158.0)	0.163
NLR	2.58 (1.76,4.59)	2.50 (1.88, 3.25)	0.715
PWR	26.80 (20.55, 31.92)	25.69 (19.38, 35.23)	0.349
