# Supplementary material for: Increased Circulating Levels of CRP and IL-6 and Decreased Frequencies of T and B Lymphocyte Subsets Are Associated With Immune-Related Adverse Events During Combination Therapy With PD-1 Inhibitors for Liver Cancer
Source: Front Oncol. 2022 Jun 8;12:906824. doi: 10.3389/fonc.2022.906824 (PMC9232255; doi:10.3389/fonc.2022.906824)
Supplement: Supplementary Table 3 — Univariate binary logistic regression analysis for risk factors of all grade and grade3/grade4 irAEs. BCLC, Barcelona Clinic Liver Cancer; M, metastasis; PVTT, portal vein tumor thrombus; TACE, transcatheter arterial chemoembolization; CRP, C-reactive protein; IL-6, interleukin-6;AFP, Alpha‐Fetoprotein; ALB, albumin; TBIL, total bilirubin; ALT, alanine aminotransferase; AST, aspartate aminotransferase; CHE, cholinesterase; LDH, lactate dehydrogenase; PTA, prothrombin activity; WBC, white blood cell; ANC, absolute neutrophil count; LYM, absolute lymphocytes; AMC, absolute monocyte count; PLT, platelets; NLR, neutrophil-lymphocyte ratio; PLR, platelet -lymphocyte ratio; PWR, platelet-white blood cell ratio. [file Table_3.pdf]

Table S3 Univariate binary logistic regression analysis for risk factors of all grade and grade3/grade4 irAEs.

	**Univariate analysis for all grade irAEs**			**Univariate analysis for G3/G4 irAEs**		
	p value	Odds ratio	95% CI	p value	Odds ratio	95% CI
Age(year)						
<58	1.000	0	0	1.000	-	-
≥58	0.558	1.333	0.509-3.495	0.621	0.757	0.251-2.281
Sex						
Male	1.000	-	-	1.000	-	-
Female	0.115	0.312	0.073-1.33	0.716	1.316	0.299-5.788
Diagnosed						
Hepatocellular carcinoma	1.000	-	-	1.000	-	-
Cholangiocarcinoma	0.822	0.844	0.193-3.697	0.979	0.978	0.178-5.375
Child-Pugh stage						
A	1.000	-	-	1.000	-	-
B	0.720	0.839	0.321-2.194	0.834	0.889	0.295-2.676
BCLC						
B	1.000	-	-	1.000	-	-
C(PVTT)	0.757	1.227	0.355-4.791	0.513	1.650	0.368-7.391
C(M)	0.395	0.536	0.723-9.323	0.965	0.9650	0.192-4.839
Treatment						
Other combination treatment	1.000	-	-	1.000	-	-
Sintilimab+Lenvatinib	0.003	6.588	1.874-23.163	0.049	8.242	1.006-67.532
TACE treatment	0.144	2.596	0.543-9.118	0.322	1.898	0.534-6.748
Immunotherapy as systemic						
First line	0.192	3.385	0.543-21.113	0.473	2.292	0.239-22017
Second line	0.215	4	0.447-35.788	0.482	2.500	0.194-32.194
Third line	0.931	1.091	0.153-7.802	0.760	0.667	0.049-9.022
Fourth line	1.000	-	-	1.000	-	-

AFP						
<400(ng/mL)	1.000	-	-	1.000	-	-
≥400(ng/mL)	0.800	0.881	0.331-2.345	0.512	1.450	0.478-4.403
CRP						
<8.2(mg/L)	1.000	-	-	1.000	-	-
≥8.2(mg/L)	0.033	3.077	1.092-8.671	0.102	2.860	0.812-10.069
IL-6						
<18(pg/mL)	1.000	-	-	1.000	-	-
≥18(pg/mL)	0.027	3.143	1.142-8.651	0.325	1.753	0.573-5.362
Total lymphocyte						
<1260(count/μL)	1.000	-	-	1.000	-	-
≥1260(count/μL)	0.795	1.144	0.414-3.166	1.000	1.000	0.302-3.308
T lymphocyte						
<883(count/μL)	1.000	-	-	1.000	-	-
≥883(count/μL)	0.446	1.477	0.542-4.025	0.545	1.149	0.436-4.814
CD4+ T lymphocyte						
<477(count/μL)	1.000	-	-	1.000	-	-
≥477(count/μL)	0.799	0.878	0.324-2.384	0.230	2.127	0.621-7.291
CD8+ T lymphocyte						
<347(count/μL)	1.000	-	-	1.000	-	-
≥347(count/μL)	0.799	0.878	0.324-2.384	0.545	1.449	0.436-4.814
B lymphocyte						
<114 (count/μL)	1.000	-	-	1.000	-	-
≥114 (count/μL)	0.437	0.667	0.240-1.854	0.543	0.688	0.206-2.297
ALB						
<35 (g/L)	1.000	-	-	1.000	-	-
≥35 (g/L)	0.693	0.824	0.314-2.157	0.621	0.757	0.251-2.281
DBIL						
<17.1(umol/L)	1.000	-	-	1.000	-	-

	≥17.1(umol/L)	0.817	1.143	0.369-3.54	0.969	0.974	0.267-3.557
TBIL							
	<34.2(umol/L)	1.000	-	-	1.000	-	-
	≥34.2(umol/L)	0.376	1.733	0.512-5.863	0.757	1.231	0.330-4.596
ALT							
	<40 (U/L)	1.000	-	-	1.000	-	-
	≥40 (U/L)	0.052	2.732	0.990-7.536	0.284	1.836	0.605-5.572
AST							
	<40 (U/L)	1.000	-	-	1.000	-	-
	≥40 (U/L)	0.087	2.528	0.875-7.305	0.424	1.674	0.473-5.927
ALP							
	<135 (U/L)	1.000	-	-	1.000	-	-
	≥135 (U/L)	0.693	0.824	0.314-2.517	0.621	0.757	0.251-2.281
γ-GT							
	<50 (U/L)	1.000	-	-	1.000	-	-
	≥50 (U/L)	0.534	1.441	0.455-4.563	0.424	0.600	0.171-2.099
CHE							
	<5000(U/L)	1.000	-	-	1.000	-	-
	≥5000(U/L)	0.926	1.050	0.377-2.922	0.728	0.809	0.245-2.675
LDH							
	<245 (U/L)	1.000	-	-	1.000	-	-
	≥245 (U/L)	0.397	0.659	0.251-1.730	0.187	0.465	0.149-1.452
PT							
	<13(s)	1.000	-	-	1.000	-	-
	≥13(s)	0.251	0.565	0.213-1.498	0.297	1.833	0.587-5.725
PTA							
	<75(%)	1.000	-	-	1.000	-	-
	≥75(%)	0.605	1.299	0.483-3.495	0.438	0.625	0.191-2.048
WBC							

	<4.0(10^9/L)	1.000	-	-	1.000	-	-
	≥4.0(10^9/L)	0.741	0.842	0.305-2.324	0.622	1.350	0.410-4.448
ANC (10^9/L)							
	<2.0(10^9/L)	1.000	-	-	1.000	-	-
	≥2.0(10^9/L)	0.895	0.983	0.395-2.450	0.571	0.713	0.221-2.299
LYM (10^9/L)							
	<0.8(10^9/L)	1.000	-	-	1.000	-	-
	≥0.8(10^9/L)	0.856	0.904	0.305-2.679	0.784	0.843	0.249-2.855
AMC (10^9/L)							
	<0.4(10^9/L)	1.000	-	-	1.000	-	-
	≥0.4(10^9/L)	0.452	1.462	0.544-3.929	0.894	0.928	0.305-2.817
RBC (10^{12}/L)							
	<3.97(10^{12}/L)	1.000	-	-	1.000	-	-
	≥3.97(10^{12}/L)	0.720	1.192	0.456-3.118	0.834	1.125	0.374-3.386
Hemoglobin							
	<128(g/L)	1.000	-	-	1.000	-	-
	≥128(g/L)	0.720	1.192	0.456-3.118	0.442	1.548	0.508-4.714
PLT							
	<124(10^9/L)	1.000	-	-	1.000	-	-
	≥124(10^9/L)	0.510	0.722	0.275-1.900	0.524	0698	0.232-2.107
PLR							
	<121.5	1.000	-	-	1.000	-	-
	≥121.5	0.895	0.938	0.359-2.450	0.442	1.548	0.508-4.14
NLR							
	<2.63	1.000	-	-	1.000	-	-
	≥2.63	0.558	1.333	0.509-3.495	0. 946	1.038	0.345-3.127
PWR							
	<26.5	1.000			1.000	-	-
	≥26.5	0.924	1.048	0.401-2.740	0.946	1.038	0.345-3.127

Abbreviation: BCLC, Barcelona Clinic Liver Cancer; **M,** metastasis; **PVTT,** portal vein tumor thrombus; **TACE,** transcatheter arterial chemoembolization; **CRP**, C-reactive protein; **IL-6**, interleukin-6;**AFP**, Alpha-Fetoprotein; **ALB**, albumin; **TBIL**, total bilirubin; **ALT**, alanine aminotransferase; **AST**, aspartate aminotransferase; **CHE**, cholinesterase; **LDH**, lactate dehydrogenase; **PTA**, prothrombin activity; **WBC**, white blood cell; **ANC**, absolute neutrophil count; **LYM**, absolute lymphocytes; **AMC**, absolute monocyte count; **PLT**, platelets; **NLR**, neutrophil-lymphocyte ratio; **PLR**, platelet -lymphocyte ratio; **PWR**, platelet-white blood cell ratio.
